# Socially-Assigned Race, Healthcare Discrimination and Preventive Healthcare Services

**DOI:** 10.1371/journal.pone.0064522

**Published:** 2013-05-21

**Authors:** Tracy MacIntosh, Mayur M. Desai, Tene T. Lewis, Beth A. Jones, Marcella Nunez-Smith

**Affiliations:** 1 Department of Emergency Medicine, School of Medicine, Yale University, New Haven, Connecticut, United States of America; 2 Department of Chronic Disease Epidemiology, School of Public Health, Yale University, New Haven, Connecticut, United States of America; 3 Robert Wood Johnson Foundation Clinical Scholars Program, School of Medicine, Yale University, New Haven, Connecticut, United States of America; 4 Department of Epidemiology, School of Public Health, Emory University, Atlanta, Georgia, United States of America; 5 Section of General Internal Medicine, Department of Internal Medicine, School of Medicine, Yale University, New Haven, Connecticut, United States of America; 6 Global Health Leadership Institute, Yale University, New Haven, Connecticut, United States of America; University of Washington, United States of America

## Abstract

**Background:**

Race and ethnicity, typically defined as how individuals self-identify, are complex social constructs. Self-identified racial/ethnic minorities are less likely to receive preventive care and more likely to report healthcare discrimination than self-identified non-Hispanic whites. However, beyond self-identification, these outcomes may vary depending on whether racial/ethnic minorities are perceived by others as being minority or white; this perception is referred to as socially-assigned race.

**Purpose:**

To examine the associations between socially-assigned race and healthcare discrimination and receipt of selected preventive services.

**Methods:**

Cross-sectional analysis of the 2004 Behavioral Risk Factor Surveillance System “Reactions to Race” module. Respondents from seven states and the District of Columbia were categorized into 3 groups, defined by a composite of self-identified race/socially-assigned race: Minority/Minority (M/M, n = 6,837), Minority/White (M/W, n = 929), and White/White (W/W, n = 25,913). Respondents were 18 years or older, with 61.7% under age 60; 51.8% of respondents were female. Measures included reported healthcare discrimination and receipt of vaccinations and cancer screenings.

**Results:**

Racial/ethnic minorities who reported being socially-assigned as minority (M/M) were more likely to report healthcare discrimination compared with those who reported being socially-assigned as white (M/W) (8.9% vs. 5.0%, p = 0.002). Those reporting being socially-assigned as white (M/W and W/W) had similar rates for past-year influenza (73.1% vs. 74.3%) and pneumococcal (69.3% vs. 58.6%) vaccinations; however, rates were significantly lower among M/M respondents (56.2% and 47.6%, respectively, p-values<0.05). There were no significant differences between the M/M and M/W groups in the receipt of cancer screenings.

**Conclusions:**

Racial/ethnic minorities who reported being socially-assigned as white are more likely to receive preventive vaccinations and less likely to report healthcare discrimination compared with those who are socially-assigned as minority. Socially-assigned race/ethnicity is emerging as an important area for further research in understanding how race/ethnicity influences health outcomes.

## Introduction

Race is widely-recognized as a primarily social, not biological, construct. Generally in health services and outcomes research, race/ethnicity is measured as respondent self-identification. However, race/ethnicity is also ascribed to individuals by others in social interactions, referred to as socially-assigned race. One's self-identification may or may not be the same as his/her socially-assigned race. Social assignment may be a largely unrecognized determinant of observed racial/ethnic differences in healthcare outcomes.

Differences in healthcare outcomes between patients who self-identify as racial/ethnic minority and those who self-identify as white are widely-recognized [1], [2]. However, the broad demographic classifications currently used (i.e. White, Black/African American, Asian, American Indian/Alaska Native and Native Hawaiian/Pacific Islander and Hispanic), may obscure differences within racial/ethnic groups where there is often variability in phenotypic characteristics. Thus, re-examining and expanding the definitions of racial/ethnic categories is one recommended way to enhance the quality of health services research and delivery [3], and isolate factors associated with racial/ethnic healthcare disparities that may be obscured by the current categorization strategy [1], [3]. A novel study recently found self-identified racial/ethnic minority individuals who reported being perceived in societal interactions as white had higher levels of self-reported health compared with racial/ethnic minority individuals who reported being perceived by society as minority [4]. This intriguing new area of research suggests that race as it is perceived by others or socially-assigned race, in addition to self-identified race/ethnicity, may be associated with key health and healthcare outcomes. Notably, this prior work concluded being socially-assigned as non-Hispanic white conveyed an advantage in health outcomes regardless of individual self-identification. It is unknown whether this previously observed advantage of being socially-assigned as non-Hispanic white extends beyond health status to areas of persistent healthcare inequity such as receipt of preventive health services.

Patients who self-identify as racial/ethnic minorities underutilize recommended preventive health services such as age-appropriate vaccinations [5]–[10] and disease screening [11]–[17]. These racial/ethnic inequities remain despite adjusting for insurance coverage [16], socioeconomic status [11], [12], [15], [16], often raising the question of whether the experience of healthcare discrimination may be an important contributor. Patient-reported healthcare discrimination has already been shown as independently associated with limited healthcare utilization, self-reported quality of care, low adherence to care plans and poor health outcomes [18]–[23].

The Institute of Medicine identified closing the healthcare utilization gap and eliminating any contribution of healthcare provider bias to observed racial/ethnic healthcare inequities as two priorities for reducing racial/ethnic disparities in healthcare [1]. Recognizing race/ethnicity as a complex phenomenon that takes into account both self-identification and social interactions with individuals and institutions [24], we sought to first examine the agreement between self-reported race and self-report of socially-assigned race, and then characterize its association with both reported healthcare discrimination and self-reported receipt of preventive healthcare services. We hypothesized that self-identified racial/ethnic minority respondents who report being socially-assigned as minorities would report higher rates of healthcare discrimination when compared with: 1) racial/ethnic minority respondents who report being socially-assigned as non-Hispanic white and 2) self-identified non-Hispanic white respondents. We further hypothesized that self-identified racial/ethnic minority respondents who report being socially-assigned as non-Hispanic white would have higher rates of self-reported recommended preventive health service utilization compared with self-identified racial/ethnic minority respondents who report being socially-assigned as minorities. In addition, we expected that rates of utilization would be similar between self-identified minorities socially-assigned as non-Hispanic white and self-identified non-Hispanic whites.

## Methods

### Sample and Data Collection

We used data from the optional “Reactions to Race” module and the standard core sections on demographics, immunizations and preventive healthcare screening from the 2004 Behavioral Risk Factor Surveillance System (BRFSS), an annual, national, cross-sectional, random-digit dialing telephone survey coordinated by the United States' Centers for Disease Control and Prevention (CDC) [25]. The Reactions to Race module has been described elsewhere [4], and underwent iterative cognitive testing, field and pilot testing prior to use. The 2004 database was selected because it was the year with the greatest number of states fielding this optional module. The participating states included Arkansas, Colorado, Delaware, Mississippi, Rhode Island, South Carolina, and Wisconsin, as well as the District of Columbia. Almost all participants (99.8%) from states using the “Reactions to Race” module reported both self-identified race and socially–assigned race, and the final analysis excluded only 59 individuals who were missing data for either of these variables. The response rates for the participating states varied between 38.6% for Rhode Island to 62.7% for Colorado, with an overall response rate of 49.8%, consistent with typical BRFSS response rates [26]. The BRFSS data are publicly available data collected by the CDC, accessible at http://www.cdc.gov/brfss/, and ethical approval by individual institutions is not required.

### Independent Variables

The primary independent variable of interest was composite race, comprised of respondent self-identified race/ethnicity and self-reported socially-assigned race/ethnicity. Respondent self-identified race was dichotomized as either non-Hispanic white or racial/ethnic minority; the latter included black/African American, Asian, Native Hawaiian/Pacific Islander, and American Indian/Alaska Native, multiracial and “other.” All respondents who identified their ethnicity as Hispanic or Latino were included in the self-identified racial/ethnic minority category. To assess socially-assigned race/ethnicity, respondents were asked, “How do other people classify you in this country?” and all responses other than non-Hispanic white were re-categorized as being socially-assigned as minority. Racial/ethnic groups were dichotomized into non-Hispanic white and minority in order to ensure adequate category sizes for comparison. Our final analysis categorized eligible respondents into three groups, indicating how they self-identified/socially-assigned: Minority/Minority (M/M), Minority/White (M/W) and White/White (W/W). A small percentage of respondents self-identified as non-Hispanic white but reported being socially-assigned as minority (White/Minority (W/M), n = 248, 0.73%). Our primary hypotheses involved comparing healthcare outcomes between the M/M, M/W and W/W groups. Because the W/M group comprised less than 1% of the sample, this group was excluded from the present analysis.

The covariates in our analysis were age, sex, marital status, employment status, high school completion, annual household income, and health insurance status.

### Dependent Variables

#### Healthcare Discrimination

To assess racial/ethnic healthcare discrimination, respondents were asked, “Within the past 12 months when seeking health care, do you feel your experiences were worse than, the same as, or better than people of other races?” Response options were, “worse than other races,” “the same as other races,” “better than other races,” “worse than some races, better than other races,” and “only encountered people of the same race.” We collapsed responses into a three-level variable. “Worse than other races” or “worse than some, better than others” responses were classified as “yes” to healthcare discrimination. Those who reported their treatment as the same or better than other races were classified as “no” to healthcare discrimination. Respondents who did not know, or were unsure, were classified as “uncertain.” A small percentage (0.27%) of participants responded that they “only encountered people of the same race” when seeking healthcare, and were excluded from analysis.

#### Healthcare outcomes: Having a personal physician and receipt of preventive healthcare services

We also evaluated seven self-reported healthcare outcomes of interest, all categorized as “yes” or “no” binary variables for eligible respondents: (1) having a personal physician, (2) receipt of influenza vaccination within the last year if ≥65 years of age [27], (3) receipt of pneumococcal vaccination if ≥65 years of age [28], (4) breast cancer screening (received both mammogram and clinical breast exam) within the last year for women ≥40 years of age[29], (5) cervical cancer screening (Pap smear) within the last 3 years for women ≥21 years of age [29], (6) prostate cancer screening (received both prostate-specific antigen (PSA) test and digital rectal exam (DRE)) within the last year for men ≥50 years of age [29], and (7) colorectal cancer screening (fecal occult blood test (FOBT) within the last year or colonoscopy within the last 10 years) for individuals ≥50 years of age [29]. Age-appropriate early cancer detection indicators were selected based on the 2004 American Cancer Society guidelines [29].

### Data Analysis

First, we performed standard frequency analyses to describe the sample. Second, we performed bivariate analyses using the chi-square test to examine the unadjusted associations between racial/ethnic assignment groups (M/M, M/W or W/W) and sociodemographic variables, having a personal physician, perceived healthcare discrimination, and receipt of preventive healthcare services. We examined both overall associations and pairwise associations among the M/M, M/W and W/W groups. Finally, we used multivariable logistic regression modelling to assess the association between socially-assigned race/ethnicity and each healthcare outcome, adjusting for age, health insurance, marital status, education, employment, income and sex, where appropriate, to calculate odds ratios and confidence intervals for healthcare outcomes significant at the 0.05 level. Analyses were conducted with SAS software Version 9.2 [30], and SUDAAN software Release 10.0 [31], and incorporated weighting to account for sampling design.

## Results

### Sample Characteristics

The overall sample included 33,679 respondents (Table 1). The majority (78.1%) of the sample self-identified as non-Hispanic white, 15.5% self-identified as black, 4.4% self-identified as Hispanic, and 1.2% self-identified as multiracial (not presented in Table 1). Other racial/ethnic groups comprised less than 1% of the sample. The majority was married (60.1%) and employed (63.5%). About one-half was female (51.8%) and had annual household incomes greater than $35,000 (50.9%). The vast majority of respondents had completed at least high school (90.0%) and reported having health insurance (85.5%).

**Table 1:** Sample demographic characteristics of 2004 BRFSS Reactions to Race respondents.

			Racial/ethnic assignment groups			P-values for Pairwise Comparisons			
			(self-identified/socially-assigned)						
		Total^a^	White/ White^b^ (W/W)	Minority/ White^c^ (M/W)	Minority/ Minority^d^ (M/M)	Overall P-value	W/W vs. M/W	W/W vs. M/M	M/W vs. M/M
		(n = 33,679)	(n = 25,913, 76.9%)	(n = 929, 2.8%)	(n = 6837, 20.3%)				
Category	Subcategory	n(%)	n (%)	n (%)	n (%)				
**Age (years)**						<0.001	<0.001	<0.001	NS
	<35	7305 (31.6)	4957 (28.2)	268 (44.0)	2080 (43.2)				
	35–49	9838 (30.1)	7420 (30.3)	261 (26.8)	2157 (29.8)				
	50–64	9200 (22.1)	7397 (23.5)	216 (17.0)	1587 (17.6)				
	65+	7154 (16.2)	6018 (18.0)	179 (12.2)	957 (9.4)				
**Sex**						0.002	<0.001	NS	<0.001
	Female	20877 (51.8)	15760 (52.0)	527 (43.1)	4590 (52.6)				
	Male	12802 (48.2)	10153 (48.0)	402 (56.9)	2247 (47.4)				
**Married**		17684 (60.1)	14835 (64.8)	487 (55.6)	2362 (41.5)	<0.001	0.001	<0.001	<0.001
**Employed**		20015 (63.5)	15521 (64.1)	500 (61.4)	3994 (61.4)	0.012	NS	0.005	NS
**Completed High School**		30216 (90.0)	23928 (92.8)	784 (85.0)	5504 (79.4)	<0.001	<0.001	<0.001	0.004
**Annual Household Income ($)**						<0.001	<0.001	<0.001	<0.001
	<15000	3667 (8.8)	2191 (6.6)	144 (13.0)	1332 (16.8)				
	15000–35000	9099 (28.5)	6272 (25.6)	281 (31.8)	2546 (39.6)				
	>35000	16647 (50.9)	14122 (56.0)	385 (43.0)	2140 (31.4)				
	Missing	4266 (12.0)	3328 (11.8)	119 (12.1)	819 (12.7)				
**Health Insurance**		29526 (85.5)	23318 (88.5)	763 (80.1)	5445 (74.3)	<0.001	<0.001	<0.001	0.01

a Numbers may not sum to total n due to missing data.

b White/White is defined as self-identified White/socially-assigned as White.

c Minority/White is defined as self-identified Minority/socially-assigned as White.

d Minority/Minority is defined as self-identified Minority/socially-assigned as Minority.

Respondents were categorized into 3 groups, defined by self-identified race/socially-assigned race (minority or non-Hispanic white): M/M (n = 6,837, 19.0%), M/W (n = 929, 3.4%), and W/W (n = 25,913, 77.6%). There was high agreement between self-identified and socially-assigned race for white (98.5%) and black (95.6%) respondents. However, 26% of Hispanic respondents reported being socially-assigned as white, 4.7% as black and 7.4% as other. The M/W group was comprised almost exclusively of participants who self-identified as Hispanic (98.5%). The M/M group differed significantly from the M/W group on a number of sociodemographic variables (Table 1). Compared with the M/M group, the M/W group was more likely to be married (55.6% vs. 41.5%, p<0.001), more likely to have completed high school (85.0% vs. 79.4%, p = 0.004), had higher annual household incomes (p<0.001) and was more likely to have health insurance (80.1% vs. 74.3%, p = 0.01). Both groups were less likely to have health insurance compared with the W/W group (88.5% among W/W respondents, p-values <0.001). Compared with either self-identified minority group, the W/W group was significantly older (51.0±16.8 years) and more likely to be married (64.8%), to have completed high school (92.8%) and to have a higher annual household income (Table 1) (p-values <0.01). The W/W group was more likely to be employed (64.1%, p = 0.005) compared to the M/M group only, and there was no significant difference in employment rates between the M/W group and either comparison group.

### Adjusted analysis of association between socially-assigned race and healthcare outcomes

Although both W/W and M/W groups had higher odds of having a personal physician compared with the M/M group in initial analyses, this relationship was significant for only the W/W group (AOR = 1.15, 95% CI: 1.02, 1.29, Table 2). M/W respondents were more likely to receive both influenza (AOR = 1.83, 95% CI: 1.16, 2.87) and pneumococcal (AOR = 1.43, 95% CI: 0.93, 2.20) vaccinations than the M/M group, although the latter relationship was in part explained by sociodemographic variables (Table 2). Similarly, the W/W group was significantly more likely to receive either vaccination than the M/M group (influenza: AOR = 1.81, 95% CI: 1.48, 2.21; pneumococcal: AOR = 2.20, 95% CI: 1.78, 2.71). There were no statistically significant differences between the M/M and M/W groups for uptake of any of the appropriate cancer screening tests. In contrast, after adjustment, W/W women were less likely to have had appropriate breast cancer screening (AOR = 0.87, 95% CI: 0.77, 0.98) and cervical cancer screening (AOR = 0.67, 95% CI: 0.54, 0.84) compared with M/M women (Table 2). Unadjusted prostate and colorectal cancer screening odds ratios were higher for W/W respondents compared with M/M respondents; however, these relationships were attenuated in the adjusted analysis (Table 2). The M/W respondents had lower odds of healthcare discrimination compared with M/M respondents (AOR = 0.61, 95% CI: 0.39, 0.95, Table 2), after adjustment for potential confounders. W/W respondents also had lower odds of healthcare discrimination than M/M respondents (AOR = 0.27, 95% CI: 0.22, 0.33) and M/W respondents.

**Table 2:** Logistic regression analysis of the association between racial/ethnic assignment group and health-related outcomes (Minority/Minority as reference group).

	Racial/ethnic assignment groups		
	(self-identified/socially-assigned)		
Health Outcomes	Minority/Minority	Minority/White	White/White
	OR (CI)	OR (CI)	OR (CI)
Have Personal Physician			
Unadjusted OR	1.00	1.03 (0.87, 1.23)	1.67 (1.55, 1.78)
Adjusted OR a	1.00	1.07 (0.79, 1.46)	1.15 (1.02, 1.29)
Received Influenza vaccine within last 12 months			
Unadjusted OR	1.00	2.12 (1.36, 3.31)	2.26 (1.87, 2.73)
Adjusted OR a	1.00	1.83 (1.16, 2.87)	1.81 (1.48, 2.21)
Ever received pneumococcal vaccine			
Unadjusted OR	1.00	1.56 (1.01, 2.40)	2.48 (2.05, 3.00)
Adjusted OR a	1.00	1.43 (0.93, 2.20) p = 0.1	2.20 (1.78, 2.71)
Breast Cancer Screening			
Unadjusted OR	1.00	1.08 (0.80, 1.47)	1.23 (1.10, 1.38)
Adjusted OR b	1.00	0.89 (0.65, 1.21)	0.87 (0.77, 0.98)
Cervical Cancer Screening			
Unadjusted OR	1.00	1.14 (0.65, 1.98)	1.05 (0.86, 1.27)
Adjusted OR b	1.00	0.89 (0.49, 1.60)	0.67 (0.54, 0.84)
Prostate Cancer Screening			
Unadjusted OR	1.00	1.50 (0.95, 2.37)	1.40 (1.13, 1.74)
Adjusted OR b	1.00	0.93 (0.73, 1.19)	0.93 (0.73, 1.19)
Colorectal Cancer Screening			
Unadjusted OR	1.00	1.32 (0.84 2.05)	1.42 ( 1.18, 1.71)
Adjusted OR a	1.00	1.01 (0.83, 1.24)	1.01 (0.83, 1.24)
Perceived Healthcare Discrimination			
*Yes*			
Unadjusted OR	1.00	0.56 (0.36, 0.86)	0.20 (0.17, 0.24)
Adjusted OR a	1.00	0.61 (0.39, 0.95)	0.27 (0.22, 0.33)
*Uncertain*			
Unadjusted OR	1.00	1.31 (0.87, 1.97)	1.32 (1.15, 1.50)
Adjusted OR a	1.00	1.34 (0.86, 2.09)	1.24 (1.06, 1.44)

OR  =  Odds Ratio; CI  =  Confidence Interval.

p = 0.05.

a Regression Model adjusted for sex, health insurance status, marital status, education, employment status, income and age.

b Regression Model adjusted for health insurance status, marital status, education, employment status, income and age.

## Discussion

We found that U.S. adults in this study who self-identify as racial/ethnic minorities, but report being socially-assigned as non-Hispanic white, reported better healthcare outcomes compared with self-identified racial/ethnic minorities who are socially-assigned as racial/ethnic minorities. This minority/white (M/W) group was also significantly less likely to report healthcare discrimination compared with the minority/minority (M/M) group. However, the M/W group was significantly more likely to report healthcare discrimination than the white/white (W/W) group in our sample (results not shown). M/M respondents were less likely to have a personal physician or a medical home, and had lower rates of annual influenza and pneumococcal vaccinations compared with both the M/W and W/W groups. The M/W and W/W groups had similar rates of influenza immunization, and the M/W group had lower rates of pneumococcal immunization than the W/W group. These key differences in healthcare outcomes persisted after we adjusted for potential explanatory factors including health insurance, marital status, education, employment, and income.

Previously published comparisons with whites have repeatedly demonstrated lower rates of both influenza and pneumococcal vaccinations among African Americans and Hispanic Americans [5]–[10], populations disproportionately burdened with bacterial pneumonia [32] and associated, preventable hospitalizations [33]. Yet, our finding that M/W group immunization and cancer screening rates were most frequently similar to those of W/W respondents, suggests M/W represents a unique subset of racial/ethnic minority patients. These findings are consistent with earlier research demonstrating that self-identified racial/ethnic minorities who are socially-assigned as white had better self-reported overall health status compared to those who are socially-assigned as non-white [4]. Contrary to our initial hypothesis, there were no differences in prostate or colorectal cancer screening rates between groups, and, after adjustment, M/M women were more likely to receive breast and cervical cancer screening compared with W/W women. Prior studies have found no clear association between perceived discrimination and cancer screening [18], [34], [35], suggesting that although racial/ethnic minority patients experience discrimination in healthcare settings, they may participate in cancer screening programs that employ culturally-appropriate outreach strategies. In turn, immunization programs may seek to adopt some of these methods.

The concept of “white advantage” conferred to a sub-group of self-identified racial/ethnic minority individuals should be considered in our sample, and Hispanic Americans are most likely among racial/ethnic minority groups to be socially-assigned as white, thus potentially benefitting from this phenomenon. Among many of the socio-demographic indicators, including having completed high school and household income, the W/W and M/M groups were at disparate ends of the spectrum, while the M/W group consistently had intermediate proportions or mean values. Our findings of relative socioeconomic privilege among the M/W group may reflect the continued legacy of racism and skin colour prejudices that are perpetuated in the United States. For example, there is substantial evidence that among racial/ethnic minorities, darker skin pigmentation is associated with lower educational attainment, occupational status, and income [36], [37], and is adversely associated with health outcomes such as mortality risk [38], and self-reported physical health [39]. Therefore, being a minority who is perceived as non-Hispanic white may confer both socioeconomic and healthcare advantages.

Our findings that M/M respondents were almost twice as likely as M/W respondents to report having experienced healthcare discrimination in the previous year, and that M/W respondents reported healthcare discrimination more frequently than W/W respondents, likely have important implications. Previous reports have associated healthcare discrimination with poor health outcomes [22], [40], [41], and other studies have associated healthcare discrimination with delays in obtaining ordered tests and treatment, not filling prescriptions [21], and low patient satisfaction and adherence [42]. Although both self-identification and social assignment of minority status are associated with reported healthcare discrimination, our findings suggest M/W and M/M respondents represent two distinct groups. Moreover, the M/W status conveys a unique experience within the healthcare setting; rates of reported healthcare discrimination and utilization of vaccinations for the M/W group differs significantly from either the M/M or the W/W groups. Findings from the emerging field of physician implicit bias, suggesting a role for unconscious influences on decision-making, may be particularly relevant in this context [43]–[47].

Our work represents a novel inquiry into how self-identified race and socially-assigned race might influence interactions within healthcare settings. Nevertheless, there are some limitations. Although the BRFSS offered a unique opportunity to conduct this study in a large sample of U.S. adults, there are some challenges inherent to its cross-sectional design and sampling approach. First, we cannot assess causality or directionality; however, we have firmly demonstrated several significant associations which merit further exploration. Second, the study relied on the self-report of preventive health service utilization and self-report of socially-assigned race. Validation studies of self-reported immunization and cancer screening, have demonstrated that these data are generally valid, particularly within one year [48]–[50], and development and validation of self-reported racial/ethnic healthcare discrimination measures remains an area of active research [51], [52]. We also lack data on the demographic or other characteristics of healthcare providers and systems with whom respondents interacted. Still, this work has strength in the capture of divergent healthcare experiences from a broad sample of participants across a spectrum of racial/ethnic identities. Third, we selected the 2004 survey because it is the survey year with the greatest number of states using the optional “Reactions to Race” module. Although the overall response rate was typical for BRFSS, the results may only be applicable to the seven states and District of Columbia which self-selected to obtain this information from their residents. Fourth, all non-white respondents were collapsed together to create the M/W and M/M categories. Because certain minority groups enjoy better overall health status, our dichotomous categorization scheme may have attenuated the impact of socially-assigned race on marginalized racial/ethnic minorities, and it fails to demonstrate all of the inequities existing between racial/ethnic minority groups. Therefore, the differences across groups may be even greater than we observed in our analyses. Finally, our study examined multiple healthcare outcomes, thereby increasing our likelihood of a type I error, or rejecting the null hypothesis. Although there is internal consistency in our results, readers should bear this in mind.

These results suggest that in order to fully understand how race and racism may be mediating health inequities in the United States, it is important to assess not only how patients self-identify their race/ethnicity, but also how they report their socially-assigned race/ethnicity. Future studies are needed to investigate the correlation between self-reported socially-assigned race/ethnicity and race/ethnicity reported by observers and to elucidate the mechanisms by which socially-assigned race leads to biases and inequities in healthcare. Provider bias and patient experience of bias may be barriers to receipt of immunizations and merits further investigation and provider education. Understanding the factors enabling full participation in preventive care by the M/W group will allow future interventions to be designed and targeted in the most efficient ways possible.
